# Change in incidents of suicidal acts after intervention on a bridge in South Korea

**DOI:** 10.1007/s00127-024-02744-9

**Published:** 2024-08-09

**Authors:** Sangsoo Shin, Jane Pirkis, Matthew J. Spittal, Lay San Too, Angela Clapperton

**Affiliations:** Centre for Mental Health and Community Wellbeing, Melbourne School of Population and Global Health, The University of Melbourne, Parkville, VIC 3052 Australia

**Keywords:** Suicidal behaviour, Suicide, Bridge, Intervention, Means restriction

## Abstract

**Purpose:**

To investigate whether two novel interventions on a bridge – a Video Incident Detection System (VIDS) and spinning bar barriers – have an impact on suicidal behaviour on the bridge.

**Methods:**

A total of 146 suicidal acts were retrieved for analyses; 108 interventions before suicidal acts, 35 suicide deaths and 3 suicide attempts. Incident rate ratios (IRR) were calculated to estimate the change in incident rate associated with implementation of the two interventions: VIDS and the spinning bar 2-metre high barrier.

**Results:**

The results of the Poisson regression showed that the rate of suicide deaths, after installation the VIDS, did not change significantly (IRR: 1.23, 95% Confidence Interval [95% CI]: 0.59–2.56), although the rate of intervened suicidal acts increased (IRR: 2.40, 95% CI: 1.65–3.47). The results showed that subsequent spinning bar installation resulted in a decrease in the incident rate of intervened suicidal acts (IRR: 0.37, 95% CI: 0.25–0.57) as well as suicide deaths (IRR: 0.23, 95% CI: 0.07–0.71). Comparison of the period when both interventions were in place with the period with no interventions indicated a reduction in suicide deaths (IRR: 0.28, 95% CI: 0.10–0.82), but no change in intervened suicidal acts (IRR: 0.90, 95% CI: 0.59–1.38).

**Conclusion:**

The rate of suicide death decreased after the installation of the spinning bar barrier but not after the implementation of VIDS alone. Our findings reinforce that restricting access to means is a highly effective way of preventing suicide on bridges and that spinning bars may be a helpful way to design barriers.

## Introduction

Suicides in public places often involve jumping from heights. An empirically verified way of preventing suicide at these sites is the restriction of access to means [1–3]. Examples of this intervention include barriers, fences, horizontal nets or even road closures that limit access to the site.

Numerous suicide intervention studies on bridges have concentrated mainly on means restriction, and the mechanism to prevent suicide has been straightforwardly described – it “buys time” [4]. Buying time may give others an opportunity to intervene [5]. The person might be thwarted by a third party before, or on, reaching the edge of the jumping spot. Alternatively, buying time may give the suicidal person themselves pause to reconsider their actions [6]. The person might step back from the jumping site themselves, even without help from others, as they were not able to easily reach the jumping point. The way in which means restriction may work in terms of giving others the opportunity to intervene is fairly obvious. The way in which it might work for the individual themselves is less well understood. Some commentators have argued that it may be particularly effective in circumstances where the individual is acting impulsively [7] or is ambivalent [6], giving them time to rethink their actions and choose a preferable course. This implies that the person is on the verge of a suicidal act at the point they are stopped. However, means restriction methods may go beyond buying time and have a preventive effect for people at earlier stages of suicidal acts, potentially acting as a more general deterrent.

Although studies have tended to focus on means restriction, there may be a continued demand for alternative interventions for various reasons. Factors such as budget limitations or community concerns regarding the aesthetics or preservation of historic sites could drive the demand for different suicide prevention measures. In addition to restriction of access to means, increasing the likelihood of intervention by a third-party is another key suicide prevention strategy. As such, video surveillance systems, either in isolation or in conjunction with other measures, is emerging as a viable strategy for preventing suicides in public places [8].

A systematic review highlighted that Closed-Circuit Television and Video (CCTV) surveillance systems could contribute to suicide prevention in three ways: (1) identification of risk factors for suicide, (2) understanding suicide after an attempt, and (3) as part of an intervention [8]. Namely, CCTV can be integrated into suicide prevention interventions by detecting potential suicide attempts as early as possible and triggering rapid responses. It is important to note that studies on the advanced systems designed to track individuals at risk more promptly, were insufficient to demonstrate their effectiveness on suicidal behaviours. The absence of scientific proof regarding the efficacy of such interventions may hinder their adoption at other locations.

A dual two-lane highway toll bridge over a bay in South Korea, opened to traffic on 1 July 2008. The bridge is 1.7 km long and the clearance below (distance between sea level and bridge span) is 68 m. Suicidal behaviours became frequent on the bridge, leading the operating company to apply several preventive measures. Initial interventions included CCTVs to improve surveillance and an emergency broadcasting system to deliver warning messages by the operation control facility. These interventions were followed by a system known as the Video-based Incident Detection System (hereafter, the VIDS) and 1-metre high spinning bars over existing 1-metre guard rails. The VIDS had been originally developed for early detection of incidents on the road. Fourteen speed sensors, installed at intervals of 300-metres on the bridge, can warn the operation control team if the speed of a car is below 30 km/hour. These complement four tracking CCTV cameras connected to speed sensors which follow the vehicle and allow the operation control team to be dispatched instantly for rapid responses to unusual behaviour. The spinning bars intervention takes the form of four spinning rollers on top of the guard rails on both sides of the bridge and makes it impossible for a person to grab hold of the bar and climb up to a jumping point.

Combining datasets on suicide deaths and other suicide-related behaviours that precede suicides at a given site would be one possible way to better understand the effectiveness of interventions at different stages of suicidal risk. Such an analysis could provide insights into how these interventions work and at which stage they are most effective. However, this is difficult. Collating epidemiologic data on suicide deaths at given sites is relatively straightforward, providing that – at a minimum – data are available on cause and location of death. Figures on other suicidal acts at such sites are much harder to come by. For this reason, a relatively scant body of research has compared statistics on suicides and suicide attempts at public sites for the pre- and post-intervention periods after a means restriction intervention (usually a barrier) has been installed (e.g., [9]). However, even this study, did not deliberately focus on suicidal behaviours preceding a suicide death as a meaningful, independent point for surveillance.

Given the current demands for interventions other than restriction of access to means, and the need for understanding the exact mechanism by which other interventions might be effective, the main purpose of the current study was to examine the point at which the interventions on a bridge have an impact on suicidal acts; do they only buy time, or do they do something in addition to this? In addition, the study aimed to evaluate the effectiveness of the VIDS and the spinning bars interventions in preventing suicide. We hypothesised that deploying the two intervention measures would result in the prevention of suicide deaths and suicide related behaviours at the site.

## Methods

### Data

Counts of suicidal acts occurring on the bridge between 1 July 2008 and 31 July 2022 were obtained from the operation company responsible for overall management of the bridge. The company has collected this information on respective incidents as part of the company’s operational performance objectives related to safety, efficiency and improved service to drivers. The data collected has been regularly audited by the local government. Three different types of incidents were captured: (1) suicidal acts where intervention occurred before jumping; (2) non-fatal suicide attempts by jumping; and (3) suicide deaths by jumping. Aggregated tables of counts of each act were made available within pre-specified timeframes, so all data were anonymous.

### Design

The classification of incidents was based on discussions about uniformly agreed terms that are designed to capture suicidal behaviours in a fine-grained manner [10, 11]. However, these categories were modified slightly. For all categories intent was determined by police after investigation.

- **Suicidal acts where intervention occurred before jumping**: This was used as an umbrella term indicating suicidal behaviours on the bridge that were identified by the operation control team before any suicide attempt was made. These included recognisable, unusual behaviours, preparatory suicidal behaviours (e.g., parking the car with a plan to jump, wandering around the bridge in a state of ambivalence, or climbing the fence). For analysis, only acts where the person had a clear intention to die by suicide, were included in this category.
- **Non-fatal suicide attempts by jumping**: These were defined as incidents where the person jumped off the bridge with intent to die and survived.
- **Suicide deaths by jumping**: These were defined as incidents where the person jumped off the bridge with intent to die and did not survive, based on the investigation by the police. No suicides by other methods than jumping from the bridge were reported during the study period.

The overall data collection period was split into three periods, classified by the time when each new intervention of interest was implemented.

- **Period 1 (No intervention period)**: 2,375 days (1 July 2008 to 31 December 2014) when the original 1-metre high rail and CCTV were put in place on the bridge. These interventions were not intended solely for suicide prevention.
- **Period 2 (VIDS only)**: 1,065 days (1 January 2015 to 30 November 2017).
- **Period 3 (VIDS and Spinning bars)**: 1,704 days (1 December 2017 to 31 July 2022).

### Statistical analysis

Incident rate ratios (IRR) were generated to estimate the change in incident rate associated with installation of each of the two later interventions: the VIDS and the spinning bars. First, rates of intervened acts and of suicidal acts were calculated separately for the pre and post periods by dividing the number of incidents by the time period, Second, these rates were then divided to produce the IRR for the periods of the interventions: the VIDS and the spinning bars. This is the most common method to evaluate the effectiveness of interventions in similar suicide prevention studies (e.g., a meta-analysis [1], a Cochran review [12]), and the best available method given the data available to the researchers. The reference period differed for different models. As the population size of the area of interest was stable over the observation period, we did not include the population size in our rate calculations. Point estimates of IRRs were presented with 95% confidence intervals (95% CI). All analyses were performed in R version 4.0.5 using the fmsb package.

## Results

In total, 146 incidents were observed during the study period. Table 1 provides a breakdown of these by incident type and period. In total, there were 108 suicidal acts where intervention occurred before jumping, three non-fatal suicide attempts by jumping, and 35 suicides by jumping. The latter two figures equate to a case fatality rate of 92.1%. For the rate of suicide deaths, 0.008 incident per day was reported in Period 1, 0.01 in Period 2, and 0.002 in Period 3. There was an increase over time in the proportion of total incidents accounted for by suicidal acts where intervention occurred, from 61.1% in Period 1, to 79.3% in Period 2, to 85.3% in the Period 3.

**Table 1:** Type of interventions and descriptive statistics for incident type by period

	Period 1	Period 2	Period 3
	1 July 2008 to 31 December 2014 (2,375 days)	1 January 2015 to 30 November 2017 (1,065 days)	1 December 2017 to 31 July 2022 (1,704 days)
Interventions	1) 1-metre high rail 2) CCTVs	1) 1-metre high rail 2) CCTVs **3) VIDS**	**1) 2-metres high spinning bar rail** 2) CCTVs 3) VIDS
Intervened suicidal acts before attempt (A)	33	46	29
Survived suicide attempts (B)	< 5	< 5	< 5
Suicide deaths (C)	20	11	< 5
Total suicidal acts (A + B + C)	54	58	34
[A / (A + B + C)] X 100	61.1%	79.3%	85.3%

Note 1) CCTV refers to closed-circuit television

Note 2) VIDS refers to Video Incident Detection System

**Fig. 1:**
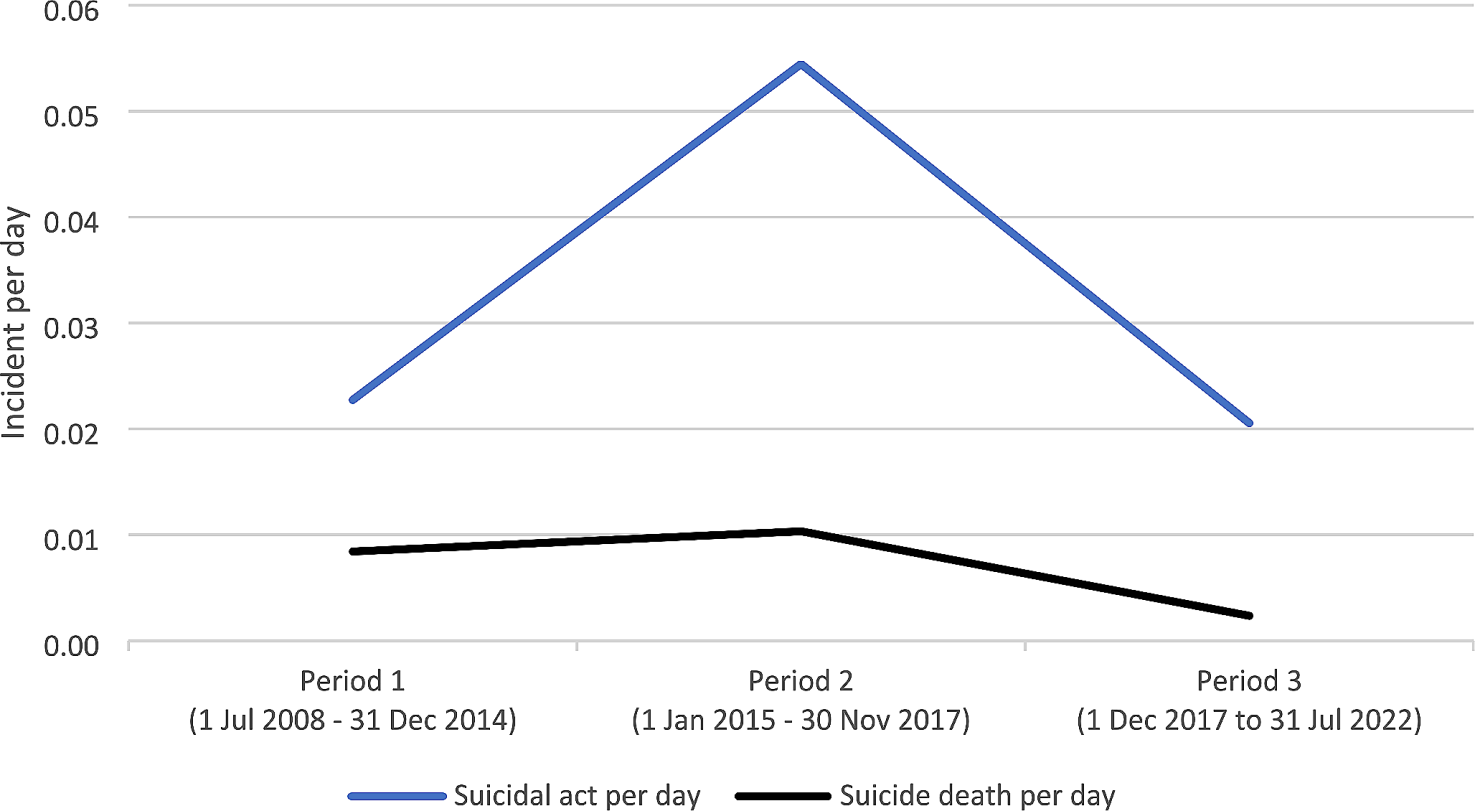
Incident rates by type of incident and period

Figure 1 presents the incident rates for suicidal acts where intervention occurred before jumping, and for suicide deaths by jumping (no equivalent figures are presented for non-fatal suicide attempts by jumping because of their low numbers). Table 2 presents incident rate ratios for comparisons of these incident types between periods. The incident rates for suicidal acts where intervention occurred before jumping were 0.023 in Period 1, 0.054 in Period 2, and 0.021 in Period 3 (IRR Period 2 vs. Period 1: 2.40, 95% CI: 1.65–3.78; IRR Period 3 vs. Period 1: 0.90, 95% CI: 0.59–1.38; IRR Period 3 vs. Period 2: 0.37, 95% CI: 0.25–0.57). The incident rates for suicide deaths by jumping were 0.008 in Period 1, 0.010 in Period 2, and 0.002 in Period 3 (IRR Period 2 vs. Period 1: 1.23, 95% CI: 0.59–2.56; IRR Period 3 vs. Period 1: 0.28, 95% CI: 0.10–0.82; IRR Period 3 vs. Period 2: 0.23, 95% CI: 0.07–0.71).

**Table 2:** Incident rate ratios by incident type and period

			Intervention period	
			Period 2	Period 3
Comparison period	Period 1	Intervened suicidal acts	**2.40 [1.65–3.47]**	0.90 [0.59–1.38]
		Suicide deaths by jumping	1.23 [0.59–2.56]	**0.28 [0.10–0.82]**
	Period 2	Intervened suicidal acts	N/A	**0.37 [0.25–0.57]**
		Suicide deaths by jumping	N/A	**0.23 [0.07–0.71]**

Bold font indicates statistical significance at the ρ < 0.05 level.

## Discussion

The results of this study clearly indicate that restricting access to means on a bridge by installing a suicide barrier led not only to substantial decreases in suicide deaths by jumping, but also in the onsite presence of people at risk of suicide for whom intervention needed to occur. There was also an increase in intervention activities for individuals at risk of suicide through the operation of an advanced system to detect unusual behaviours. However, its effectiveness in reducing suicide deaths did not reach statistical significance in Period 2 compared to Period 1.

Throughout the whole study period, there were a total of 35 suicide deaths from the bridge, which equates to 2.5 deaths per year; 3.3 per year in the periods before installation of the spinning bars (Period 1 + Period 2). The number of suicide deaths in the pre-intervention period at the site included in this study was lower than the corresponding figure of 5.9 deaths from the 13 sites where means restriction was applied in a meta-analysis [1]. There were 0.9 suicide deaths per year from the bridge after installation, equating to a decrease in the death rate by 74%. This result is consistent with previous studies indicating that barriers can be extremely effective in preventing suicide, even at sites where suicides are relatively less frequent [7, 13].

There were significant reductions in the number of suicidal acts where intervention occurred before jumping in Period 3 relative to Period 2. This suggests a more comprehensive understanding of how this intervention might work, including the concept of “buying time”, but not limited to that concept. One interpretation of this finding is that the spinning bars (potentially in combination with the VIDS) were not only effective in thwarting suicide attempts when they were imminent (i.e., “buying time”), but also that they acted as a cognitive deterrent, resulting in fewer people approaching the site with suicidal intent in the first place. Although suicide by violent methods sometimes occurs with little pre-thought and planning [14], suicide by methods with lower expected lethality tend to be more impulsive [15]. In the case of suicides from the bridge in our study, a relatively high degree of planning might be required. This is because, as noted above, access to the highway toll bridge is limited to vehicles. This suggests that the suicidal person requires certain, extra preparation (including driving a car to and then onto the bridge), and that consequently there might be opportunities for them to change their mind in the preparatory phase.

Understanding the impact of the VIDS requires careful thought. There was a significant increase in the number of intervened suicidal acts before jumping in Period 2 (when the VIDS was introduced) relative to Period 1. One explanation for this is that there may have been a genuine increase in the numbers of people who were on the bridge behaving in a manner that suggested that they might have been at imminent risk, potentially because of the site becoming more prominent because of the VIDS. This iatrogenic effect is unlikely for at least two reasons. Firstly, the devices associated with the VIDS are unlikely to be recognised as a part of a suicide prevention intervention; their appearance, resembling speed checkers and CCTVs on other roads, would not suggest to drivers that the bridge is a site associated with suicide. Secondly, the increase in intervened suicidal acts prior to jumping predated installation of the VIDS by one year, beginning in 2014, which suggests that the increase may be associated with external factors. In fact, one such external factor may be an increase in the numbers of vehicles crossing the bridge; this increased by around 53% in 2014 compared with in 2013. A systematic review on the train/subway suicide indicated that suicidal acts increased with the number of passengers [16]; the so-called familiarity hypothesis.

An alternative explanation is that more interventions occurred as a result of the VIDS. The significant increase in suicidal acts for which an intervention was made before jumping in Period 2 (compared with Period 1) was accompanied by no significant increase in suicide deaths. This might suggest that the VIDS contributed to curbing suicide deaths by allowing unusual behaviours to be detected prior to the person carrying out a suicide attempt, even though the level of effectiveness of the VIDS was not able to be quantified. In fact, the percentage of intervened suicidal acts increased to 79% in Period 2 from 61% in Period 1. The installation of the VIDS might have allowed intervention by staff after the detection of several behaviours that otherwise would not have been detected, and these behaviours might have reflected a broader range of crisis levels. Individuals who engage in some suicidal behaviours, albeit not all, may be more effectively stopped through intervention before they progress to a fatal stage of behaviour. This observation suggests the possibility of a “buying time” effect of non-structural intervention, which involves spotting unusual movements onsite as early as possible.

The high case fatality rate found in our study underscores the importance of prioritising appropriate interventions before individuals carry out their act. The case fatality rate for jumping from this 68-metre-high bridge was 92.1% which aligns with prior research indicating that bridge height is positively associated with case fatality rate; the case fatality rate was more than 95% for jumping from the 75-metre Clifton Suspension Bridge in the United Kingdom [17], 75% for jumping from bridges of around 55 m in Pittsburgh, the United States [18], and 18% from bridges lower than around 15 m, also in Pittsburgh [18].

The findings not only corroborate the association between bridge height and risk of suicide death, but suggest that rescue strategies following a suicide attempt from a high bridge are unlikely to effectively reduce the overall number of suicides. Instead, strategies that prevent people from making a suicide attempt must be prioritised.

### Strengths and limitations

Although other studies have examined suicide prevention interventions on bridges [1], this is the first study to evaluate the effectiveness of spinning bars, and application of new advanced surveillance technology, the VIDS. It is also the first to examine the cumulative impact of more than one intervention, by progressively examining changes in suicidal phenomena as different interventions were introduced. Few other studies have considered multiple different examples of suicidal behaviour, with most considering suicide deaths by jumping only. Given most previous research assessing the effectiveness of means restriction has concentrated on sites where suicides are frequent, another strength of this study is that it shows that means restriction was effective in a place where suicide deaths were relatively sparse. This study also makes a unique contribution by exploring what the results mean for the mechanisms by which different interventions might work. The data used in this study very likely represents all suicide incidents from the bridge, which is only accessible by vehicle, providing a means to track vehicles. Also, the data used covers the whole period since the establishment of the bridge. Finally, it is the first study to examine suicide prevention interventions on an Asian bridge.

Despite these strengths, the study had several limitations. One notable limitation was the reliance on data supplied by the company responsible for the bridge, which restricted the ability to evaluate the potential for displacement effects or substitution to other fatal methods. Displacement to other bridges or substitution to other fatal methods might have happened to some extent, but we were unable to explore these issues using the available dataset that was limited to only the one bridge. As is typical in studies on quasi experimental designs, it was not possible to consider a baseline period when no interventions had been put in place; in Period 1, a 1-metre high rail and CCTV cameras had already been installed. Further, it was not possible to account for potential confounders (e.g., a potential change in pattern of epidemiology of suicide during the Covid-19 pandemic) and how these might have played out across the different periods. The findings from this study should be generalised with caution; as noted, the bridge has some unique features, including lack of pedestrian access.

## Conclusion

This study further reinforces that restricting access to means is a highly effective way of preventing suicide on bridges. However, it goes beyond this, suggesting that spinning bars may a helpful way to design barriers, and that restricting access to means may work (either in isolation or in combination with measures to increase the likelihood of intervention by a third party) not only because it “buys time” but also because it may act as a more general deterrent.

## Data Availability

No datasets were generated or analysed during the current study.

## References

[1] Pirkis J et al (2015) Interventions to reduce suicides at suicide hotspots: a systematic review and meta-analysis. Lancet Psychiatry 2(11):994–1001 26409438 10.1016/S2215-0366(15)00266-7

[2] Sinyor M et al (2017) Did the suicide barrier work after all? Revisiting the Bloor Viaduct natural experiment and its impact on suicide rates in Toronto. BMJ Open 7(5):e015299 28634260 10.1136/bmjopen-2016-015299 PMC5734210

[3] Berman AL, Athey A, Nestadt P (2022) Effectiveness of restricting access to a suicide jump site: a test of the method substitution hypothesis. Inj Prev 28(1):90–92 34417196 10.1136/injuryprev-2021-044240

[4] Daigle MS (2005) Suicide prevention through means restriction: assessing the risk of substitution - A critical review and synthesis. Accid Anal Prev 37(4):625–632 15949453 10.1016/j.aap.2005.03.004

[5] Bennewith O, Nowers M, Gunnell D (2011) Suicidal behaviour and suicide from the Clifton Suspension Bridge, Bristol and surrounding area in the UK: 1994–2003. Eur J Pub Health 21(2):204–208 20630909 10.1093/eurpub/ckq092

[6] Cox GR et al (2013) Interventions to reduce suicides at suicide hotspots: a systematic review. BMC Public Health, 13 10.1186/1471-2458-13-214 PMC3606606 23496989

[7] Pelletier AR (2007) Preventing suicide by jumping: the effect of a bridge safety fence. Inj Prev 13(1):57–59 17296691 10.1136/ip.2006.013748 PMC2610560

[8] Onie S et al (2021) The Use of closed-circuit television and video in suicide Prevention: Narrative Review and future directions. Jmir Mental Health, 8(5) 10.2196/27663 PMC8140380 33960952

[9] Ueda M, Sawada Y, Matsubayashi T (2015) The effectiveness of installing physical barriers for preventing railway suicides and accidents: evidence from Japan. J Affect Disord 178:1–4 25770476 10.1016/j.jad.2015.02.017

[10] Silverman MM (2016) *The international handbook of suicide prevention*, in *The international handbook of suicide prevention*, R.C. O’Connor and J. Pirkis, Editors. John Wiley & Sons. pp. 11–35

[11] De Leo D et al (2021) International study of definitions of English-language terms for suicidal behaviours: a survey exploring preferred terminology. Bmj Open, 11(2) 10.1136/bmjopen-2020-043409 PMC7875264 33563622

[12] Okolie C et al Means restriction for the prevention of suicide by jumping. Cochrane Database Syst Reviews, 2020(2). 10.1002/14651858.CD013543 PMC7039710 32092795

[13] Reisch T, Michel K (2005) Securing a suicide hot spot: effects of a safety net at the Bern Muenster Terrace. Suicide Life-Threatening Behav 35(4):460–467 10.1521/suli.2005.35.4.460 16178698

[14] Cheah D, Schmitt G, Pridmore S (2008) Suicide, misappropriation and impulsivity. Aust N Z J Psychiatry 42(6):544–546 18465382 10.1080/00048670802050611

[15] Rimkeviciene J et al (2015) Personal stigma in suicide attempters. Death Stud 39(10):592–599 26086667 10.1080/07481187.2015.1037972

[16] Too LS et al (2014) The socio-environmental determinants of railway suicide: a systematic review. BMC Public Health, 14 10.1186/1471-2458-14-20 PMC3922773 24405530

[17] Bennewith O, Nowers M, Gunnell D (2007) Effect of barriers on the Clifton suspension bridge, England, on local patterns of suicide: implications for prevention. Br J Psychiatry 190:266–267 17329749 10.1192/bjp.bp.106.027136

[18] Brice JH et al (2013) Epidemiology of Low-Level Bridge Jumping in Pittsburgh: a 10-Year study. Prehospital Emerg Care 17(2):155–161 10.3109/10903127.2012.722179 23148589

